# Keloid Formation and Any Skin Complications in Patients Treated With Isotretinoin and Undergone Any Skin‐Related Procedures

**DOI:** 10.1111/jocd.16680

**Published:** 2024-11-20

**Authors:** Raha Latifaltojar, Arash Pour Mohammad, Azadeh Goodarzi

**Affiliations:** ^1^ School of Medicine Iran University of Medical Sciences Tehran Iran; ^2^ Department of Dermatology, Rasool Akram Medical Complex Research Development Center (RCRDC), School of Medicine Iran University of Medical Sciences (IUMS) Tehran Iran

**Keywords:** acne, hyperpigmentation, isotretinoin, keloid, laser, scar

## Abstract

**Background:**

Isotretinoin is widely used for moderate to severe acne vulgaris. Despite its broad application, isotretinoin carries a risk of permanent scarring and keloid formation following various skin procedures. As a result, a delay of at least 6–12 months after completing or discontinuing isotretinoin treatment is commonly recommended before undergoing skin procedures.

**Aims:**

This study aims to evaluate the necessity of delaying skin procedures performed concurrently with or soon after isotretinoin treatment at different dosages in patients with acne vulgaris, based on the dermatological side effects associated with combination therapy.

**Methods:**

A literature search was conducted using PubMed, Scopus, Web of Science, and Embase databases for original studies up until June 2023.

**Results:**

A total of 34 eligible studies, including 1563 patients treated with isotretinoin, were reviewed to assess the timing of various skin procedures, safe dosages, and potential adverse effects, such as keloid formation and persistent hyperpigmentation which were reported in a few cases.

**Conclusions:**

Based on our review, there is insufficient evidence to support delaying laser hair removal, ablative fractional lasers, nonablative fractional lasers, superficial to medium‐depth chemical peels, manual dermabrasion, cutaneous surgeries, fractional microneedling radiofrequency, microdermabrasion, and dermaroller treatments. However, fully ablative lasers, mechanical dermabrasion, and ablative radiofrequency procedures are not recommended during isotretinoin use. Further studies are needed to establish the safety and optimal interval for these procedures. For all skin procedures, especially more aggressive and deeper ones, a lower dose of isotretinoin is recommended.

## 1 Introduction

Acne vulgaris is a chronic inflammatory skin disease that can involve every part of the body with a high concentration of pilosebaceous units [1, 2]. It has been known as the eighth most prevalent disease in the world with a prevalence of 9.4% in 2010 [2]. Acne has been found to be the most common skin condition in the United States, which influences up to 50 million American every year. Some patients exhibit severe inflammatory acne which further results in severe acne scars. It has been reported that acne especially with scar formations, can lead to psychosocial and social disorders such as depression, anxiety, suicidal ideation, and physical impacts. Hence, there is a need for quick and effective treatment to prevent and decrease these consequences [3, 4, 5].

Isotretinoin (13‐cis‐retinoic acid, Accutane), an FDA‐approved drug, has been used widely since the early 1980s for moderate to severe acne vulgaris [6, 7]. Despite of wide application of isotretinoin in the treatment of acne, there are some reports of an increased risk of permanent scar and keloid formation in some literature after laser therapy (Argon laser, 585 nm pulsed dye laser), dermabrasion and chemical peeling procedures. Based on these reports, it is recommended to delay such skin procedures for at least 6–12 months after completion or disruption of isotretinoin treatment [8, 9, 10, 11, 12]. Some literature reported keloid formation after the usage of isotretinoin without any skin procedures [13]. Moreover, there are no reports of keloid formations soon after the usage of isotretinoin or concomitant use of this drug and skin procedures in recent literature [14].

The aim of this study is (i) To determine the need to delay skin procedures concomitant or soon after discontinuation of isotretinoin treatment in patients with acne vulgaris, (ii) We tried to determine the dermatological side effects of combination therapy with isotretinoin and different procedures (iii) The safe dosage and the safe interval between drug treatment and different skin procedures if it was possible based on original available literature.

## 2 Methods

### 2.1 Search Strategy and Study Selection

We searched PubMed, Scopus, Web of Science, and Embase for original English literature published until June 2023 and we could access their full texts. The search syntaxes of different databases have been provided in the Supporting Information S1. The case reports and studies with fewer than five participants, publications based on books and congress, and which did not mention or target dermatological side effects of combination therapy, were excluded from our study. After screening and eligibility assessment of literature with two different reviewers and the resolution of disagreements by a third reviewer, 34 literature were finally included. We classified our findings into eight major topics: LHR (Laser Hair Removal), Laser for Resurfacing treatment, Chemical peeling, Dermabrasion, Surgeries, Radiofrequency interventions, Dermaroller and Microdermabrasion, and combination therapy (of different procedures). We also assessed publications by using the Oxford Centre for Evidence‐Based Medicine's Levels of Evidence [15].

### 2.2 Quality Assessment

After removing duplicate studies, the articles were screened separately by two authors in terms of title and abstract, and the difference of opinions was decided by a third author. Then these articles were re‐examined by two authors by reviewing the entire text of the articles, and the differences of opinion were examined by a third person. At this stage, the articles were finally included or excluded from the study according to the inclusion and exclusion criteria.

### 2.3 Data Synthesis

All of the transient and permanent dermatological side effects (e.g., keloid formation, delayed wound \healing, and pigmentation) associated with concomitant use of isotretinoin and different skin procedures, the dosage of the drug, and the interval between skin procedures and isotretinoin consumption, extracted with two different researchers. Then, they inserted the data extraction form.

## 3 Results

**FIGURE 1:**
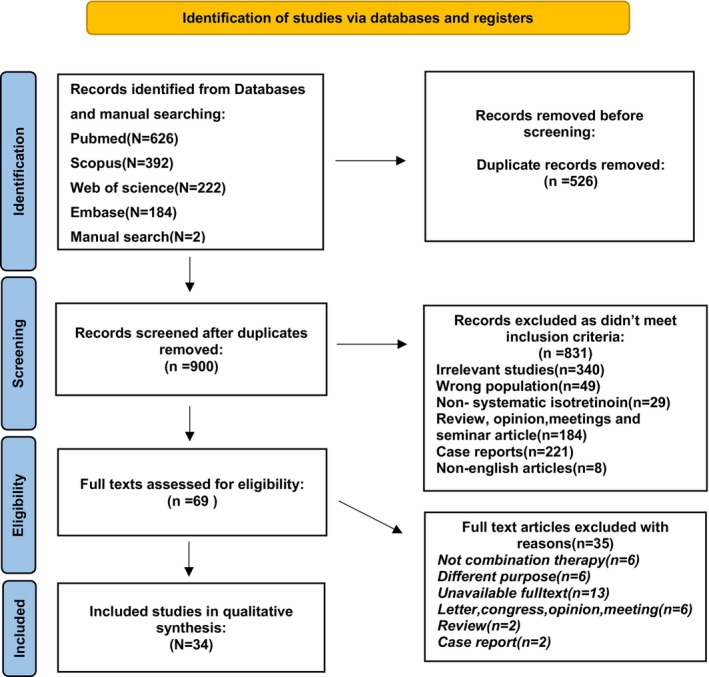
PRISMA flow diagram of the study.

34 eligible literature with a total sample size of 1563 patients treated concomitantly with isotretinoin and different procedures, were included in our study. The process of reviewing as a PRISMA flowchart is shown in Figure 1 [16]. Of these 17 were clinical trials, seven were retrospective cohorts, two were prospective cohorts, and eight were case series. Summary of study characteristics are summarized in Table 1.

**TABLE 1:** Summary of the reviewed studies on the use of different dermatological procedures in the setting of isotretinoin treatment.

Study	Study design	Control	Total Sample size	Fitzpatrick skin type	Isotretinoin dose	Isotretinoin use	Intervention	Transient side effects	Permanent sequelae	Follow up	Level of evidence
*Laser hair removal*											
Khatri, 2004 [17]	Case series	—	7	2, 3	20–80 mg/d	Concomitant	Diode 810 nm	Transient erythema, bulla(resolved)	—	1 week, 1 month later	4
Cassano et al. 2005 [18]	Case series	—	6	2, 3	0.5–1 mg/kg/d, decreased to 0.3–0.5 mg/kg/d	Concomitant	Diode 810 nm	Transient erythema, sparse dotted crusting	—	Within 4 years period	4
Khatri and Garcia, 2006 [19]	Case series	—	6	2	40–80 mg/day	Concomitant	Long‐pulse flash lamp	Transient erythema	—	1 and 3 weeks, 1 month later	4
Khatri, 2009 [20]	Case series	—	11	3, 4, 5	0.5 mg/kg/d	Concomitant	Long‐pulse Nd: YAG 1064 nm*	Erythema and perifollicular edema, crusting and transitory PIH in the abdominal area(1p)	—	4–10 and 12 weeks later	4
Bs et al. 2014 [21]	Retrospective cohort	55p: (5p for LHR) did not receive isotretinoin	110 (5P for LHR)	—	10–40 mg/d	Concomitant	Nd:YAG 1064 nm Diode 980 nm, IPL	—	—	6, 12, 18, and 24 weeks later	3
Mahadevappa et al. 2016 [22]	Prospective cohort	—	183 (12 for LHR)	2, 3, 4, 5, 6	0.5 mg/kg/d	Concomitant or prior to	Nd:YAG 1064 nm Diode, IPL	—	—	June 2012 to May 2013	2
Guduk et al. 2021 [23]	Retrospective cohort	52p**: LHR alone	104	2, 3, 4	10–50 mg/d 0.2–0.8 mg/kg/d	Concomitant	Alexandrite, diode, and Nd:YAG laser	Crusting, temporary hypopigmentation(positive history of atopic eczema)	—	—	2
*Ablative lasers(resurfacing and treatment)*											
Bs et al. 2014 [21]	Retrospective cohort	55p:(25P for CO_2_ laser): did not receive isotretinon	110 (25P for CO_2_ laser)	—	10–40 mg/d	Concomitant	Fractional CO_2_ laser	Erythema and edema, mild crusting	—	6, 12, 18, and 24 weeks later	3
Kim et al. 2014 [24]	Case series	—	20	3, 4	10–40 mg/d	Concomitant or 4 weeks prior to	Ablative fractional CO_2_ laser	Tiny crusts surrounded by slight‐to‐moderate erythema	—	6 months to 4 years	2
Mahadevappa et al. 2016 [22]	Prospective cohort	—	183 (58 for Ablative lasers)	2, 3, 4, 5, 6	0.5 mg/kg/d	Concomitant or prior to	Fractional Co_2_ laser, Conventional Full‐face Co_2_ laser, Fractional Er: YAG laser	Transient erythema, post inflammatory hyperpigmentation after Conventional ablative Co_2_ laser prolonged redness, PIH, and flare of acne after Er: YAG laser	—	June 2012 to May 2013	2
Chiwo González, F. S, 2018 [25]	Clinical trial	The elbow side of the arm	9	—	—	4 weeks prior to	CO_2_ Ablative Fractional Laser 1060 nm	—	—	1/5 and 6 weeks after surgery	3
*Nonablative lasers(resurfacing and treatment)*											
Yoon et al. 2014 [26]	Clinical trial	18p: only infrared fractional laser	53 (35p as combination therapy)	2, 3, 4, 5	10 mg/d	Concomitant	Erbium‐doped 1550 nm NAFL	Transient erythema	—	After each treatment	2
Mahadevappa et al. 2016 [22]	Prospective cohort	—	183 (1 case of nevus of Hori)	2, 3, 4, 5, 6	0.5 mg/kg/d	Concomitant or prior to	Q‐switched Nd: YAG laser	Transient erythema	—	June 2012 to May 2013	2
Saluja et al. 2017 [27]	Clinical trial	Untreated side of the patient's face	10	1, 3	Cumulative dose:125–325 mg/kg	Within 1 month prior to	Erbium‐doped 1550 nm NAFL	Transient erythema and edema	—	7 days, 4 weeks, 6 months later	2
Xia et al. 2018 [28]	Clinical trial	Untreated side of the patient's face	18	2, 3, 4	10 mg/d (0.14–0.23 mg/kg/d)	Concomitant	1550‐ nm Er: glass fractional laser	Transient erythema and edema Dry mouth and cheilitis, dry eye	—	3, 6, 9 months later	2
Gao et al. 2020 [29]	Retrospective cohort	15p: isotretinoin only 15p: NAFL only	60 (15p as combination therapy)	3, 4	1 mg/kg/d for the first 2–4 weeks and then 0.5 mg/kg/d	Concomitant	1565 nm NAFL	Transient erythema and edema, scab and hyperpigmentation, cheilitis	—	1 month after the final session	2
Sarac et al. 2021 [30]	Clinical trial	20p: did not receive isotretinoin	40(20p as a combination therapy)	2, 3, 4	—	Immediately before	577 nm pro‐yellow laser	Transient erythema	—	4 weeks later	4
Liu et al. 2021 [31]	Clinical trial	33p: did not receive isotretinoin	67(34 as a combination therapy)	—	10 md/BD	Concomitant	5‐aminolevulonic acid photodynamic therapy(633 nm)	Transient erythema and pustules, pigmentation	—	4th, 6th, 8th, and 12th weeks, and 6th months	2
Ibrahim et al. 2021 [32]	Clinical trial	23p: isotretinoin only(0.5 mg/kg/d)	46 (23p as a combination therapy)	—	0.25 mg/kg/d	Concomitant	Pulsed dye laser	Transient erythema, dryness of lips and eyes	—	3 and 6 months later	2
Li et al. 2021 [33]	Clinical trial	23p: isotretinoin only	47(24p as a combination therapy)	3, 4	0.5–0.75 mg/kg/d	Concomitant	420 nm IPL	Transient erythema, blister(1p), Dryness or facial skin irritation, peeling lips	—	At 6th and 12th week and 2 months later	2
Li et al. 2022 [34]	Clinical trial	Untreated side of the patients face	33	2, 3, 4	10–20 mg/d (0.13–0.36 mg/kg/d)	Concomitant	500–600 nm DPL	Transient erythema and edema, dry cheilitis and skin, eye dryness	—	4 weeks after the last session	2
Sapra et al. 2022 [35]	Case series	—	187	—	40 mg/d(184p) 10 mg/d(2p) 80 mg/d(1p)	Concomitant or within 6 month prior to	Multiplex pulsed dye and Nd:YAG laser	Eczema, erythema, dry lips, skin, eyes(8p), bruising, flushing(6p) Cheilitis, hyperpigmentation(2p)	Keloid(1p:as a result of acne itself)	902.7 ± 870.9 days	4
Xue et al. 2023 [36]	Clinical trial	Untreated side of the patients face	24	3, 4, 5	10–20 mg/d 0.12–0.22 mg/kg/d	Concomitant	Fractional picosecond 1064 nm Nd: YAG laser(FxPico)	Transient erythema and edema, Pinpoint bleeding, cheilitis, dryness of skin and eyes	—	2nd and 3rd months	2
Xue et al. 2023 [37]	Clinical trial	20p: did not receive isotretinon	44(24p as a combination therapy)	3, 4, 5	10–20 mg/d 0.12–0.22 mg/kg/d	Concomitant	Fractional picosecond 1064 nm Nd: YAG laser(FxPico)	Transient erythema and edema, Pinpoint bleeding, cheilitis, dryness of skin and eyes	—	1st, 2nd, 3rd months	2
*Chemical peeling*											
Kar et al. 2013 [38]	Clinical trial	30p:isotretinoin only	60(30p as a combination therapy)	—	20 mg/d	Concomitant	20% SA peeling	Dryness of lips	—	Every 2 weeks for 16 weeks	2
Mahadevappa et al. 2016 [22]	Prospective cohort	—	183 p (79p for peeling)	2, 3, 4, 5, 6	0.5 mg/kg/d	Concomitant or prior to	GA(55p), SA(10p), TCA(1p), combination(13p) peeling	GA peeling: transient and 2 weeks lasting erythema Combination peeling:5 days‐lasting erythema	Facial Keloid(1p:after GA peeling; also on truncal zone)	June 2012 to May 2013	2
Bs et al. 2021 [39]	Retrospective cohort	13p: topical treatment and chemical peeling only	60(47p as a combination therapy)	—	10–20 mg/d (0.5 mg/kg/d)	Concomitant	SMP, GA peeling, modified Jessner's peel	Transient erythema, visible desquamation	Persistent hyperpigmentation(1p in both groups)	After the last session of peelings	2
Dixit et al. 2022 [40]	Clinical trial	28p: isotretinoin only	58(30p as a combination therapy)	—	20 mg/d (0.5 mg/kg/d)	Concomitant	20% SA and 10% mandelic acid peeling	Cheilitis, dryness, desquamation, itching, mild acne flare	—	0–30, 30–60, 60–90 days and after 4 months	2
Ye et al. 2022 [41]	Clinical trial	The untreated side of the patient's face	29	2, 3, 4	0.2–0.4 mg/kg/d	Concomitant	SSA peeling	Transient erythema, exfoliation, hyperpigmentation, dryness of skin and lips	—	2nd, 4th,6th,10th weeks	2
*Dermabrasion*											
Roenigk et al. 1985 [42]	Case series	—	9	—	0.5 or1 mg/kg/d	Concomitant or prior to	Mechanical full‐face dermabrasion (diamond/wire brush)	Flare of acne, milia	—	Up to 2nd month	3
Bagatin et al. 2010 [43]	Case series	—	7	—	10 to 40 mg/d	Concomitant	Manual dermabrasion (diamond fraise)	Transient erythema and crust	—	7, 30, 60, 90, and 180 days later	2
Rafiq et al. 2019 [44]	Clinical trial	79 female and 36 male: dermabrasion only	232(117p as a combination therapy)	—	20 mg/d	Concomitant	Manual dermabrasion (diamond)	Transient erythema, desquamation, dryness, stinging	—	45 days later	2
*Surgical procedures*											
Mahadevappa et al. 2016 [22]	Prospective cohort	—	183 (22p: cold steel surgery, 1p:electrosurgery, 1p:cryosurgery)	2, 3, 4, 5, 6	0.5 mg/kg/d	Concomitant or prior to	Microneedling, skin biopsy, subcision, punch elevation of scars, excision of skin lesion, and wisdom tooth extraction, electrosurgery, cryosurgery	Pigmentation after 7th session of microneedling(1p)	—	June 2012 to May 2013	2
Tolkachjov et al. 2017 [45]	Retrospective cohort	82p: did not receive isotretinoin	285(203 p as a combination therapy)	Not mentioned exactly (1–3)	Not mentioned exactly	More than 2 years prior to till concomitant	Incisional surgeries	Wound infection, excessive bleeding, wound dehiscence, maceration, postoperative bleeding	—	Till 2 years later	3
Yahyavi et al. 2020 [46]	Clinical trial	154: did not receive isotretinoin	303(149p as a combination therapy)	—	20 mg/d (0.3 mg/kg/day)	Concomitant	Rhinoplasty	Cheilitis, xerosis, headache	—	1, 3, 6, 12 months later	2
*Radiofrequency procedures*											
BS et al. 2014 [21]	Retrospective cohort	55:(13p for FMRF) did not receive isotretinoin	110 (13p for FMRF)	—	10–40 mg/d	Concomitant	Microneedling radiofrequency	Transient erythema and edema, pigmentation in patient of each group	—	6, 12, 18, and 24 weeks later	2
Mahadevappa et al. 2016 [22]	Prospective cohort		183(1 patient for radiofrequency)	2, 3, 4, 5, 6	0.5 mg/kg/d (cumulative dose of 4000 mg in this patient)	Concomitant or prior to	Radiofrequency ablation of compound nevi on the face	—	Facial keloid		2
Hasan et al. 2023 [47]	Clinical trial	20p: isotretinoin only	40(20p as a combination therapy)	—	0.5–1 mg/kg/d	Concomitant	Automated radiofrequency microneedling	—	—	4th, 8th, and 12th weeks, 6th month	2
*Dermaroller and microdermabrasion*											
BS et al. 2014 [21]	Retrospective cohort	55:(12p for dermaroller) did not receive isotretinoin	110 (12p for dermaroller)	—	10–40 mg/d	Concomitant	Dermaroller	Transient erythema and edema	—	6, 12, 18, and 24 weeks later	2
Mahadevappa et al. 2016 [22]	Prospective cohort	—	183 (18p for microdermabrasion)	2, 3, 4, 5, 6	0.5 mg/kg/d	Concomitant or prior to	Microdermabrasion‐aluminum	—	—	June 2012 to May 2013	2
*Combination therapies*											
Picosse et al. 2012 [48]	Prospective cohort	—	10	2, 3, 4, 5	Accumulated dose = 122–161 mg/kg	1–3 months prior to	Jessner's solution combined with 35% TCA followed by Manual dermabrasion	Transient erythema, pigmentation(2P)	—	6 months later	2
Gao et al. 2020 [29]	Retrospective cohort	15p: isotretinoin only 15p: NAFL only	60(15p as a combination therapy)	2, 3	1 mg/kg/d for the first 2–4 weeks and then 0.5 mg/kg/d	Concomitant	1565 nm NAFL with pricking blood therapy	Transient erythema and edema, scab and hyperpigmentation, cheilitis	—	1 month after the final session	2
Kim et al. 2020 [49]	Retrospective cohort	28p: did not receive isotretinoin	71(43p as a combination therapy)	2, 3, 4	20 mg/d	Concomitant or within 3 weeks prior to	FMRF + AFL	Transient erythema and edema	—	3 months later	3
Kim et al. 2022 [50]	Retrospective cohort	44p: isotretinoin only (Cumulative dose = 4535.0 ± 2350 mg)	126(82p as a combination therapy)	2, 3, 4	Cumulative dose = 3378.1 ± 1871.4 mg	Concomitant or within 1 month prior to	PDL and/or FMRF ± AFL	Transient erythema dryness of mouth, skin, eyes	—	12 months	2

Abbreviations: AFL, ablative fractional CO_2_ laser; CO_2_, carbon dioxide; DPL, delicate pulsed light; Er: YAG, erbium yttrium aluminum garnet; FMRF, Fractional Microneedle Radiofrequency; GA, glycolic acid; IPL, intense pulsed light; LHR, laser hair removal; NAFL, nonablative fractional laser; Nd: YAG, neodymium‐doped yttrium aluminum garnet; P, patient; PDL, Pulsed dye lase; PIH, post inflammatory hyperpigmentation; SA, salicylic acid; SMP, salicylic acid‐mandelic acid peel; SSA, supramolecular salicylic acid; TCA, trichloracetic acid.

### 3.1 LHR (Laser Hair Removal)

LHR has been widely used since 1996 after showing the effect of normal‐mode ruby laser pulses on delaying hair growth [51]. In considering of LHR mechanism of action which is based on selective photothermolysis and targeting hair follicle's melanin without touching collagen, we assumed that it should be safe even in patients on isotretinoin [52].

Seven literatures consisting of four case series [17, 18, 19, 20], two retrospective cohorts [21, 23], and one prospective cohort [22] with a level of evidence of 2–3 with a total of 99 patients treated with combination therapy of isotretinoin and laser and light‐based hair removal, were included in this category. Dermatological side effects were reported as below:

Transient erythema after laser sessions as we expected was reported in many studies [17, 18, 19, 20].

Bulla was reported in one patient after diode 810 nm laser with concomitant use of isotretinoin 20–80 mg/d that resolved without any intervention. According to the author's opinion, this was probably because of insufficient cooling during the procedure [17].

Transient crusting was reported in three different studies in a total of seven patients after diode (810 nm) [18], Long‐pulsed Alexandrite (755 nm) [23], and long‐pulsed Nd:YAG (1064 nm) [20] which was healed completely within a few days to weeks.

In one patient, 7 months after completing the isotretinoin therapy and the last treatment session with the highest fluency of Nd:YAG laser, crusting developed into transitory hyperpigmentation in the abdominal area which resolved spontaneously in 3 months [20].

Temporary hypopigmentation was reported in one patient after mixed wavelengths of 810 and 940 nm diode occurred after prolonged erythema. This patient had a history of atopic dermatitis and hypopigmentation in untreated areas [23]. Based on the fact that none of the studies have reported any permanent side effects such as delayed wound healing, keloid formation, or pigmentation alterations we can consider that the procedure can safely be conducted among patients on isotretinoin [17, 18, 19, 20, 21, 22, 23].

Both low doses and high doses (from 0.2 mg/kg/d to 80 mg/d) have been used in these studies. Therefore, the safety of the concomitant use of LHR and isotretinoin can be considered independent of the dose.

LHR treatment was concomitant or soon after the completion of the isotretinoin treatment for one to a maximum of 6 months before the start of the intervention. Therefore, there is no enough evidence for a 6‐month delay and it can even be done at the same time as the isotretinoin treatment.

### 3.2 Laser for Resurfacing Treatment

Acne and acne scars should be provided with sustained treatment as soon as possible to prevent long‐term outcomes [53]. Nowadays, lasers are one of the most prevalent choices for this purpose. There are ablative and nonablative lasers as well as fractionated and nonfractionated ones [54].

After case reports from the 1980s to the 1990s based on keloid formation and delayed wound healing after the use of isotretinoin concomitant with argon and PDL lasers, was recommended to delay laser procedures for at least 6 months after stopping isotretinoin treatment [9, 11].

In our review, we found 16 related literature consisting of two case series [24, 35], two retrospective cohorts [21, 29], two prospective cohorts [22, 25], and 10 clinical trials [26, 27, 28, 30, 31, 32, 33, 34, 36, 37] with a level of evidence of 2–4 in the field of resurfacing and treatment with different lasers. A total number of 6 patients were treated with isotretinoin and resurfacing lasers concomitant or soon after completion of drug treatment, and 238 patients as a control without recently isotretinoin treatment.

#### 3.2.1 Ablative Lasers

Four studies used ablative lasers as a therapy concomitant or soon after isotretinoin treatment [21, 22, 24, 25].

Transient erythema as we expected was reported after conventional ablative CO_2_ laser and ablative fractional CO_2_ laser, and also prolonged redness was reported in one patient after ablative fractional erbium‐doped yttrium aluminum garnet (Er:YAG) which was resolved by steroids within 2 weeks [21, 22, 24].

Based on two reports, transient and mild crusting was observed in patients after fractional CO_2_ laser treatments [21, 24].

Postinflammatory hyperpigmentation (PIH) was reported in one of 25 patients after fractional CO_2_ laser [21], 14 of 19 patients treated with a conventional full‐face CO_2_ laser, and one of 19 patients treated with fractional Er:YAG which were resolved within weeks to months after topical treatments [22].

Flare of acne was reported in one patient treated with fractional Er:YAG which was resolved by adapalene 0.1% gel [22].

No side effects, such as prolonged, persistent, and intense erythema and crusting, or the development of hypertrophic scars or keloid, epidermal pigmentation, and delayed wound healing mentioned as a result of ablative lasers.

Side effects were reported in different dosages of isotretinoin therefore we could not determine the exact isotretinoin dose for these patients but based on the aggressive nature of this procedure is recommended to use a lower dose of isotretinoin at the same time as ablative lasers. Ablative lasers in all of these studies were done concomitantly or within a maximum of 1‐month interval after isotretinoin treatment. Therefore, there is no need for a 6‐month delay, and according to recent studies, ablative fractional lasers (CO_2_ or Er:YAG) are safe to be done simultaneously or more reliably after a 1‐month interval, with better outcomes and lower doses of the drug which can also reduce isotretinoin side effects. However, there is not enough data for conventional full‐face ablative CO_2_ lasers to determine the safety and safe interval for this procedure. Therefore, more studies should be conducted to confirm the safety of this laser concomitant with isotretinoin, however, in one related study just transient PIH was mentioned and it is recommended to use lower doses of isotretinoin in these patients [22].

#### 3.2.2 Nonablative Lasers

13 eligible studies of 10 clinical trials [26, 27, 28, 30, 31, 32, 33, 34, 36, 37], one prospective cohort [22], one retrospective cohort [29], and one case series [35] with the level of evidence of 2–4 used different nonablative lasers concomitantly or within one to 6 months after discontinuation of isotretinoin in patients.

Keloid formation was reported just in one patient treated with multiplex 585 nm PDL (pulsed dye laser) and 1064 nm Nd:YAG laser without exactly mentioned interval of isotretinoin treatment while patients in this study were treated with isotretinoin concomitantly or within 6 months before laser sessions. Researchers noted that “keloid formation is likely a result of the acne itself and not a direct result of isotretinoin or NAL therapy” [35].

Transient erythema and edema after laser sessions were reported in different studies as expected which resolved within a few hours to days [26, 27, 28, 31, 32, 33, 34, 35, 36, 37].

Different degrees of temporary and controlled dryness of skin and mucus were mentioned in eight studies [28, 29, 32, 33, 34, 35, 36, 37] which in two different comparative studies were significant differences between combination groups (NAFL plus isotretinoin) and control groups (as an isotretinoin‐only or laser‐only groups) [32, 37] and no significant differences were mentioned in one study between intervention group (IPL (intense pulsed light) plus isotretinoin) and isotretinoin‐only group even though dryness was reported more in a control group [33].

Hyperpigmentation was reported in three different studies: in one patient after NAFL treatments [29], and in two patients after multiplex Nd: YAG + PDL treatments [35] which were resolved within a few days. Also in the study of Liu et al. a small number of patients from both groups showed pigmentation after the third session of PDT (photodynamic therapy) in both the intervention group (PDT plus isotretinoin) and control group (PDT only) without significant differences in incidence [31].

Blister was reported in one patient in the study group after the first intense pulsed light (IPL) treatment, which resolved within 1 week without any treatment [33].

Flare of acne was not mentioned in any of the intervention group patients after different nonablative lasers but it has been reported in six patients treated with only isotretinoin in the Ibrahim et al. study compared with none of the patients with lower doses of isotretinoin in the intervention group (PDL + isotretinoin) [32].

More acne scar improvements in combination therapy groups with isotretinoin and nonablative lasers were mentioned with significant differences in four studies versus isotretinoin‐only treated patients [27, 29, 36, 37] and without significant differences in one study versus only infrared fractional laser‐treated patients which is probably due to the differences in the number of Laser therapy sessions [26].

The improvement of acne lesions in nine studies was significantly higher in combination therapy groups with isotretinoin and nonablative lasers than in the control groups (isotretinoin‐only or laser‐only groups) [28, 29, 31, 32, 33, 34, 35, 36, 37].

Also, relapse rates were reported less with significant differences in combination therapy groups than the control groups [31, 33]. Patient's satisfaction was examined in nine studies and all of them were more satisfied with the combination therapy of isotretinoin and nonablative lasers [27, 28, 29, 32, 33, 34, 35, 36, 37].

None of the mentioned side effects were permanent. One case of observed keloid also did not occur as a result of the treatment.

The precise dose of isotretinoin in the patient with keloid is not mentioned but blister was reported in higher doses of the drug (> 0.5 mg/kg/d) and PIH was seen in both high and lower doses of isotretinoin. Also in most of the studies with lower doses of isotretinoin (< 0.5 mg/kg/d), none of the unexpected side effects were seen [26, 27, 28, 34, 36, 37]. Therefore, we can conclude that it is preferred to use lower doses of isotretinoin concomitant with nonablative lasers.

Drug treatment in all of the studies was done concomitantly or within one to 6‐month intervals before laser sessions. The blister happened in the concomitant use of drug and nonablative lasers. PIH was reported in both concomitant use of isotretinoin and maybe within 6‐month intervals based on the study of Sapra and coworkers [35]. The exact interval of isotretinoin and laser sessions in the patient with keloid is not mentioned. However, in seven studies of concomitant use of drug and isotretinoin, none of the unexpected side effects were seen [26, 27, 28, 30, 34, 36, 37].

Therefore, based on more patient satisfaction, faster and better acne and acne scar improvement results from the combination therapy, we can say that the simultaneous use of nonablative lasers and isotretinoin is safe with better outcomes and there is no need for 6‐month intervals.

### 3.3 Chemical Peeling

Chemical peeling is a method of using different chemical substances to induce controlled injury to different layers of the skin including the epidermis (superficial peeling) and the dermis (medium and deep peeling) for the treatment of acne, acne scars, and PIH [55, 56].

One of the concerns for concomitant use of peeling and isotretinoin, like other skin procedures, is the formation of keloids and scars after this procedure especially medium to deep peeling [57]. Due to the case report of permanent hyperpigmentation and scarring after superficial peeling with glycolic acid, more studies are needed to evaluate the safety of combination therapy of isotretinoin and different chemical peelings [12].

In our review, five related studies of three clinical trials [38, 40, 41], one prospective cohort [22], and one retrospective cohort [39] with a level of evidence of 2 were included.

Facial keloid was reported just in one patient after GA (Glycolic acid) peeling managed by intralesional steroids and also developed keloid later on the truncal zone (cumulative dose of 2100 mg of isotretinoin) [22].

Erythema as one of the side effects after peeling, was seen in different four studies. Transient erythema which subsided within minutes to hours was reported after SSA (supramolecular salicylic acid) peeling [41], GA peeling, and SMP (salicylic‐mandelic acid) peeling [22, 39].

More persistent erythema which subsided within more than 5 days to weeks and with topical treatments was mentioned in two studies after GA peeling, and combination peeling [53].

Transient desquamation was reported after SA + mandelic acid peeling [40], SSA peeling [41], and glycolic mandelic acid peels which were more in patients treated concomitantly with isotretinoin versus patients without isotretinoin treatment [40].

PIH was reported in two studies: in the study of Ye et al. after SSA peeling was seen as transient consequence [41], Bs et al. after SMP mentioned more persistent (> 2 weeks) which insignificant differences were seen compared to the patients not treated with isotretinoin as a control group [39].

Dryness of skin and lips were mentioned in four studies all of them were transient [38, 39, 41, 58]. In the study of BS et al. dryness was seen more often with SMPs than GA or modified Jessenrs peeling in both isotretinoin and nonisotretinoin‐treated groups [39].

Improvement of acne and acne scars was reported significantly higher in combination therapy than isotretinoin or chemical peeling alone in four studies [38, 39, 40, 41].

Persistent side effects as a keloid were seen in one patient who developed keloid at the distant site postprocedure. Hence, we are not able to say that it exactly happens as a result of GA peeling, it was probably idiosyncratic. As a persistent PIH in one patient, no significant differences between the two groups were seen in the comparative study of BS et al. Based on these studies, combination treatment with superficial and moderate peeling is probably safe. However, according to Gerber's case report [12] of permanent scars and hyperpigmentation, more studies are needed to reach certainty.

In all the studies reviewed, except for one study that did not specify the dose (range of 10–40 mg/kg/d) [22], patients were treated with low‐dose isotretinoin (< 0.5 mg/k/d). Therefore, lower doses of isotretinoin are recommended.

Mentioned keloid and PIH were seen in concomitant use of isotretinoin and chemical peeling but in other studies, no persistent side effects have been reported concomitantly with the isotretinoin. Based on our review concerning more improvement and satisfaction rate of combination therapy, there is no need to delay chemical peeling after isotretinoin treatment but more clinical trials are recommended to ensure the safety of concomitant use of chemical peeling and isotretinoin and to determine the safe interval.

### 3.4 Dermabrasion

Dermabrasion is a procedure that can be done with different devices in both mechanical and manual techniques. During this intervention, the epidermis layer to the end of the papillary dermis and even less into the reticular dermis are involved and the ability to regenerate the skin remains [58]. Many case reports from 1985 to 1994, mentioned delayed wound healing and keloid formation in the setting of concomitant mechanical dermabrasion and isotretinoin treatment. After the concerns of these side effects, it was advised to delay these procedures for 6 months to 1 year within discontinuation of isotretinoin [8, 9, 10].

Three related studies of two case series [42], and one clinical trial [44] with a level of evidence of 2–3 were included in our review.

Erythema was mentioned as a consequence of dermabrasion in two studies all of them were transient and resolved within days to months [43, 44].

Transient desquamation (scaling) was reported in two different studies after manual dermabrasion as was expected [43, 44].

Dryness as a consequence of dermabrasion was reported just in one study which resolved without sequelae. In this study of Rafiq et al. based on different genders, erythema, desquamation, and dryness were seen more in the female group treated with isotretinoin versus females were not treated with isotretinoin and similar outcomes were seen between male groups [44].

Other mentioned dermatologic side effects were Millia and flare of acne in the study of Roenigk et al. after mechanical dermabrasion. The author mentioned that the flare of acne was probably a result of stopping isotretinoin too soon [42].

No serious and permanent complications such as keloid formation, pigmentation alteration, and delayed wound healing from manual dermabrasion in two different studies and mechanical dermabrasion in one study have been reported.

The difference between these studies and the case reports of keloids is in the usage of lower doses of isotretinoin compared to the higher doses in the previous studies (> 40 mg/kg/d). Therefore, it can be concluded that it is better to perform dermabrasion with lower doses of isotretinoin.

About the safe interval between drug treatment and manual dermabrasion in two studies included, concomitant use of isotretinoin is relatively safe and does not lead to delay in wound healing or keloid formation. Therefore, concomitant use of isotretinoin and manual dermabrasion seems to be safe and comes with better results based on the study of Rafiq et al. [44].

But considering the case reports of mechanical dermabrasion and more severity of this procedure, it is better to wait at least until the drug is cleared and also consider lower dosages of the drug, but clearly, more studies are needed to conclude the safe interval and safe dosage of isotretinoin in this point.

### 3.5 Surgery/Electrosurgery/Cryosurgery

Three eligible studies of one prospective cohort [22], one retrospective cohort [45], and one clinical trial [46] with the level of evidence of 2–3 with a variety of cutaneous surgical procedures while receiving systemic isotretinoin have been reported normal wound healing without any permanent sequelae and keloid formation.

Pigmentation was seen in one patient after seven sessions of microneedling therapy which resolved later [22].

In the study of Tolkachjov et al. postoperative complications (wound infection, poor healing, excessive bleeding, and wound dehiscence) were seen in both patients undergoing isotretinoin therapy and those did not expose to the medication in the perioperative period which were not significant in both groups [45].

In the study of Yahyavi et al. dryness of lips (cheilitis) and skin was reported in a small group of patients of both groups treated with and without isotretinoin preoperatively [46].

Concomitant treatment of isotretinoin and surgical procedures such as cold steel surgeries (microneedling, skin biopsy, subcision, punch elevation of scars, excision of skin lesion, tooth extraction, rhinoplasty, mastectomy, etc), and also one session of electrosurgery and cryosurgery that were mentioned in our included studies, did not induce to abnormal wound healing or any permanent scars in any patients.

Based on the fact that no side effects were seen in the lower doses of the drug in our review, it seems that using lower doses is associated with better and safer results with higher satisfaction of patients.

Drug treatment in these studies was done concomitantly or within 6‐month intervals before laser sessions. Considering the small number of available studies (three cases), more studies are needed to conclude safe intervals and safe drug dosages for different surgeries. However, it seems that there is no need to postpone surgical interventions for 6 months, especially minimally invasive surgeries.

### 3.6 Radiofrequency Interventions

Radiofrequency interventions use electrothermal energy to penetrate specific sites of the derm instead of photothermal energy which is used by lasers. Hence, it is assumed that is chromophore dependent and can be used for all skin types [59].

Two studies of one retrospective cohorts [21] and one clinical trial [47] evaluated the safety of fractional microneedling radiofrequency (FMRF) and one study of a prospective cohort [22] evaluated radiofrequency ablation concomitant with isotretinoin treatment with a level of evidence of 2–3.

Keloid reported just in one case after radiofrequency ablation of compound nevi on the face [22].

Two studies by Bs et al. and Hassan et al. mentioned FMRF as a therapy in isotretinoin‐treated patients. Transient erythema and edema were mentioned after FMRF resolved within hours to a few days as the only consequences [21]. Pigmentation was reported in the Bs's study in one patient of both treated and not‐treated groups with isotretinoin after microneedling radiofrequency which faded within 6–8 weeks [21]. Both studies reported more improvement rates in combination therapy groups than in isotretinoin or FMRF only treated groups.

Side effects such as pigmentation were not persistent and resolved within days except keloid in one patient mentioned above. All the patients were treated with isotretinoin at the time of radiofrequency interventions. On the one hand, based on reports and more improvement in combination therapy, we can conclude that nonablative types of radiofrequency especially for more superficial interventions can be safe within isotretinoin treatment and even can be used simultaneously. On the other hand, based on the lack of evidence of ablative radiofrequency interventions and for more deep lesions, more studies are recommended to determine the safety of these procedures.

Studies used variable doses of the drug so it is not possible to make a definite decision about the prescribed dose of the drug, but also lower doses should be recommended.

### 3.7 Microdermabrasion and Dermaroller

Dermaroller and microdermabrasion as the two minimally invasive procedures were mentioned in two studies of the retrospective cohort [21] and prospective cohort [22] with a level of evidence of 2.

No side effects were mentioned after microdermabrasion and just transient erythema and edema were mentioned as a side effect of dermaroller subsided within days [21, 22].

In the study of Bs et al. significant improvements were reported as a result of dermaroller combination therapy compared to patients not treated with isotretinoin [21].

Based on the two included studies, no permanent sequelae were mentioned as a result of dermaroller and microdermabrasion. Both interventions were conducted concomitant with isotretinoin treatment and different dosages were prescribed. Therefore, due to the small number of studies conducted, it is still not possible to decide the dosage, but still, according to the studies, a lower dose of the drug is associated with fewer side effects. It seems to be safe to perform dermaroller and microdermabrasion in patients treated with isotretinoin concomitantly.

### 3.8 Combination Therapy

Four studies of three retrospective cohorts [29, 49, 50], one prospective cohort [48] with a level of evidence of 2–3 evaluated concomitant use of isotretinoin and different combinations of dermatological procedures as below.

In the study of Picosse et al. medium‐depth manual Jessner + TCA (Trichloroacetic acid) peeling followed by manual dermabrasion in patients completed their drug treatment 1–3 months before the procedure was evaluated. Erythema which subsided within days to weeks and with topical treatments was mentioned after peeling. PIH was reported in two patients which resolved within months after treatment [48].

Gao et al. evaluated the safety and efficacy of 1565 nm nonablative fractional laser in combination with pricking blood therapy (PBT) in 15 patients who received concomitant isotretinoin compared with other three groups of patients (NAFL or isotretinoin‐only groups, and NAFL + Isotretinoin group).

Different degrees of cheilitis as a result of isotretinoin, transient erythema and edema, scab, and hyperpigmentation after laser sessions were reported as expected which resolved within a few days. In addition to no permanent sequelae, more improvement in acne and acne scars and satisfaction rate were reported after this combination therapy compared to other groups [29].

In the study of Kim et al. none of the patients reported delayed wound healing or adverse events after the combined use of FMRF (fractional microneedling radiofrequency) and AFL concomitantly or within 1‐month intervals of isotretinoin. Just some degree of transient erythema and edema after treatment was reported as a consequence which resolved within hours to days. Also, more improvement in acne and acne scars was reported in the combination therapy group than FMRF plus AFL alone treated group [49].

In another study by Kim et al. the efficacy and safety of the combined use of energy‐based devices, (EBD: a combination of FMRF (fractional microneedling radiofrequency) or/and PDL (Pulsed dye laser) with/without AFL (ablative fractional laser)) and isotretinoin was discussed. Severe dryness of mouth, skin, or eyes, occurred more frequently in the isotretinoin‐only group as the control group than in the energy‐based devices group treated simultaneously with isotretinoin. Transient erythema as was expected mentioned which was resolved later.

This study reported more acne improvement with a significant difference in the EBD group than the isotretinoin‐only group despite the use of a lower cumulative dose of isotretinoin in the EBD group. Also, less acne relapse was reported in combination therapy than isotretinoin group which was not significant.

No permanent complications occurred in any of the groups with concomitant or within 1‐month intervals [50].

Based on our review, neither prolonged, persistent, and intense erythema, nor the development of hypertrophic scars or keloids occurred as a result of the combination therapies as mentioned above. All of these procedures were done concomitantly with isotretinoin treatment or within 1‐month intervals therefore we can conclude that there is no need for delaying these procedures for 6 months or above. However, more studies should be conducted to evaluate the safety and efficacy of other combined procedures. These interventions are combined and are associated with better results even with lower doses of isotretinoin. Therefore, lower doses of the drug are recommended during combined treatment.

## 4 Discussion

After reviewing the body of literature of all mentioned articles, we did not find sufficient evidence to support the current recommendation to delay Laser hair removal, Ablative fractional lasers, nonablative fractional lasers, superficial to medium‐depth chemical peelings, manual dermabrasions, cutaneous surgeries, fractional microneedling radiofrequency, microdermabrasion, dermaroller and mentioned combined procedures for patients treated concomitantly with isotretinoin or soon before these procedures and they are suggested to be safe with regards to prolonged, persistent, and intense erythema and crusting, or the development of hypertrophic scars or keloid, epidermal pigmentation, and delayed wound healing in patients treated with isotretinoin recently. However, due to keloid formation in patients who are prone to it, physicians should consider the positive history of patients in terms of keloid formation and take an informed decision.

Based on the evidence from included studies to date, fully ablative lasers, mechanical dermabrasion, and ablative radiofrequency procedures are not recommended in the setting of isotretinoin use and more studies should be conducted to evaluate the safety and determine the safe interval of these procedures in patients treated with isotretinoin.

According to many studies, the use of a lower dose of isotretinoin is associated with fewer side effects, and the results of acne and acne scar improvement were also satisfactory. Therefore in all skin procedures, especially more aggressive and deeper ones, is recommended to use a lower dose of the drug.

## 5 Conclusion

Based on the data obtained, isotretinoin treatment concomitant with the skin procedures is safe in the mentioned doses and there is no need for a 6‐month delay. However, more studies are needed in the case of fully ablative lasers, mechanical dermabrasion, and ablative radiofrequency interventions. Also, this does not mean the simultaneous use of isotretinoin in all dosages, and with all interventions, and with all patients, therefore more further studies are still needed to prove the safety.

## Conflicts of Interest

The authors declare no conflicts of interest.

## Supporting information


Supporting Information S1.

## Data Availability

The authors have nothing to report.
