# Supplementary material for: Keloid Formation and Any Skin Complications in Patients Treated With Isotretinoin and Undergone Any Skin‐Related Procedures
Source: J Cosmet Dermatol. 2024 Nov 20;24(2):e16680. doi: 10.1111/jocd.16680 (PMC11845948; doi:10.1111/jocd.16680)
Supplement: Supplementary file 1 — Supporting Information S1. [file JOCD-24-e16680-s001.docx]

**Supplementary Material:**

Search was done until seventh of the June of 2023 as mentioned below:

#1 PubMed:

isotretinoin[tiab] OR roaccutane[tiab] OR “13-cis-retinoic acid”[tiab] OR 13 cis Retinoic Acid[tiab] OR Isotretinoin Zinc Salt[tiab] OR 13-cis-Isomer[tiab] OR Isotretinoin Zinc Salt, 13 cis Isomer[tiab] OR Accutane[tiab] OR Ro 4-3780[tiab]OR Ro 4 3780[tiab]OR Ro 43780[tiab] OR "Isotretinoin"[Mesh]) AND (surger*[tiab] OR laser[tiab] OR plasty*[tiab] OR procedure*[tiab] OR graft*[tiab] OR “light-therapy”[tiab] OR ipl[tiab])

#2 web of science:

(((((((((TS=(surger*)) OR TS=(laser)) OR TS=(plasty*)) OR TS=(procedure)) OR TS=("wound healing")) OR TS=(scar)) OR TS=(graft*)) OR TS=(light-therapy)) OR TS=(ipl)) AND (((TI=(isotretinoin)) OR TI=(roaccutane)) OR TI=(absorica)) OR TI=(myorisan)laser

#3 scopus:

( TITLE ( roaccutane ) OR TITLE ( isotretinoin ) OR TITLE ( zenatane ) OR TITLE ( absorica ) OR TITLE ( myorisan ) ) AND ( TITLE-ABS-KEY ( surger* ) OR TITLE-ABS-KEY ( laser ) OR TITLE-ABS-KEY ( plasty* ) OR TITLE-ABS-KEY ( procedure ) OR TITLE-ABS-KEY ( "wound healing" ) OR TITLE-ABS-KEY ( scar ) OR TITLE-ABS-KEY ( graft* ) OR TITLE-ABS-KEY ( light-therapy ) OR TITLE-ABS-KEY ( ipl ) )

#4 embase:

(roaccutane OR isotretinoin OR zenatane OR absorica OR myorisan):ti AND (surger* OR laser OR plasty* OR procedure* OR "wound healing" OR scar OR graft* OR light-therapy OR ipl):ab,ti
